# Crotoxin Inhibits Endothelial Cell Functions in Two- and Three-dimensional Tumor Microenvironment

**DOI:** 10.3389/fphar.2021.713332

**Published:** 2021-08-04

**Authors:** Ellen Emi Kato, Luciana Araújo Pimenta, Maíra Estanislau Soares de Almeida, Vanessa Olzon Zambelli, Marinilce Fagundes dos Santos, Sandra Coccuzzo Sampaio

**Affiliations:** ^1^ Laboratory of Pathophysiology, Butantan Institute, São Paulo, Brazil; ^2^ Special Laboratory of Pain and Signaling, Butantan Institute, São Paulo, Brazil; ^3^ Institute of Biomedical Sciences, Department of Cell and Developmental Biology, University of São Paulo, São Paulo, Brazil; ^4^ Institute of Biomedical Sciences, Department of Pharmacology, University of São Paulo, São Paulo, Brazil

**Keywords:** crotoxin, endothelial cells, extracellular matrix, adhesion, migration

## Abstract

Antitumor property of Crotoxin (CTX), the major toxin from *Crotalus durissus terrificus* snake venom, has been demonstrated in experimental animal models and clinical trials. However, the direct action of this toxin on the significant events involved in neovascularization, which are essential for tumor growth and survival, has not been confirmed. This study investigated the effects of CTX on the key parameters of neovascularization in two- and three-dimensional culture models. Murine endothelial cell lines derived from thymus hemangioma (t.End.1) were treated at different concentrations of CTX (6.25–200 nM). Endothelial cell proliferation, cell adhesion, and actin cytoskeletal dynamics on laminin (10 µg/ml), type I collagen (10 µg/ml), and fibronectin (3 µg/ml) were evaluated along with the endothelial cell migration and formation of capillary-like tubes in 3D Matrigel. CTX concentration of 50 nM inhibited tube formation on 3D Matrigel and impaired cell adhesion, proliferation, and migration under both culture medium and tumor-conditioned medium. These actions were not accountable for the loss of cell viability. Inhibition of cell adhesion to different extracellular matrix components was related to the reduction of αv and α2 integrin distribution and cytoskeletal actin polymerization (F-actin), accompanied by inhibition of focal adhesion kinase (FAK), Rac1 (GTPase) signaling proteins, and actin-related protein 2/3 (Arp 2/3) complex. This study proved that CTX inhibits the major events involved in angiogenesis, particularly against tumor stimuli, highlighting the importance of the anti-angiogenic action of CTX in inhibition of tumor progression.

## Introduction

Angiogenesis is a complex process involving the formation of new blood vessels from preexisting endothelium and is regulated under both physiological and pathological conditions, by a range of anti-angiogenic and proangiogenic factors (Folkman and Haudenschild, 1980; Folkman 1995; Fierro, 2005). The overexpression of angiogenic factors and down-regulation of angiogenic inhibitors, known as the “angiogenic switch,” is particularly essential for tumor progression (Folkman, 2002; Dass et al., 2007). Besides growth factors, tumor angiogenesis is also regulated by cell-cell and cell-extracellular matrix interactions mediated by adhesion receptors like cadherins and integrins (Eliceiri and Cheresh, 1998). Intracellular signaling, based on a microenvironment-induced modulation, coordinates the different cellular functions, including proliferation, differentiation, and migration (McCarty, 2020). Integrins are transmembrane receptors for several extracellular matrix (ECM) components such as laminin, collagen, and fibronectin connecting the ECM to the cytoskeleton; integrins also mediate this signaling (Barczyk et al., 2010; Schlie-Wolter et al., 2013; McCarty, 2020). The endothelial cell-ECM interaction mediated by integrins promotes intracellular signal transduction, cytoskeleton reorganization, and alterations in cell behavior, such as stimulation of endothelial cell proliferation, migration, and invasion (Varinska et al., 2017; Viallard and Larrivee, 2017). The literature reports that endothelial cells proliferate 50–200 times faster in a tumor microenvironment and express specific molecules, which may become a pharmacological target, without affecting the integrity of healthy vessels. A single vessel can support about 100 tumor cells. Thus, destroying this structure may eradicate a considerable number of tumor cells (Dass et al., 2007).

Additionally, endothelial cells in the tumor microenvironment are genetically stable and are less likely to accumulate mutations that allow drug resistance (Boehm et al., 1997). Thus, targeting tumor neovascularization is an attractive strategy for cancer therapy (Hood et al., 2002). Several animal venoms have been identified as an alternative strategy for anticancer therapies (Varinska et al., 2017). Venoms are a complex mixture of bioactive chemical substances, mainly proteins, and peptides rich in disulfide, with several pharmacological actions, making it an efficient anticancer agent. Also, venoms exhibit specificity and possess the ability to modify their molecular targets, making them good therapeutic candidates (Chatterjee, 2018). Snake venoms are a natural source of molecules that modulate cell-ECM interaction orchestrated by integrins; two large Viper venom molecules considered integrin antagonists include disintegrins and C-type lectin proteins (Marcinkiewicz, 2013). However, phospholipases A2 (PLA2-EC: 3.1.1.4) derived from snake venoms possess antitumor activity owing to their inhibitory action against several tumor cells (Rudrammaji and Gowda, 1998; Kang et al., 2000; Rodrigues et al., 2009). The cell-cell interactions (Kang et al., 2000), along with cell adhesion and migration functions (Zouari-Kessentini et al., 2009), are not necessarily dependent on their catalytic activity (Stabeli et al., 2006).

Crotoxin (CTX) is the most abundant toxin (nearly 60% of the total venom) in *Crotalus durissus terrificus* (Laurenti, 1768) venom (CdtV). It is a heterodimeric β-neurotoxin, formed by the non-covalent association of two different subunits: an acid denominated as CA (Crotoxin A) or crotapotin and a base named CB (Crotoxin B) or phospholipase A2. Several studies have shown that CTX has immunomodulatory, anti-inflammatory, antimicrobial, analgesic, and antitumor effects (Sampaio et al., 2010; Sartim et al., 2018). Antitumor activity of CTX has been demonstrated particularly on tumor cells, in both *in vitro* and *in vivo* experimental trials, as well as in clinical trials (Rodrigues et al., 2009; Sampaio et al., 2010). CTX has shown significant regression of various tumors, particularly solid tumors, in clinical trials, besides pain relief and improvement in overall clinical status (Costa et al., 1998; Cura et al., 2002). This inhibitory activity has also been evidenced in experimental models aimed at characterizing the mechanisms involved in the antitumor effects of CTX. *In vitro* studies have highlighted that CTX incubation in different tumor cell lines induces significant inhibition on the proliferation of murine and human tumor cells, including decreased expression of receptors for growth factors, cytotoxic activity, mitochondrial membrane potential, necrosis, and autophagy (Newman et al., 1993; Rudd et al., 1994; Donato et al., 1996; Kang et al., 2000; Papo and Shai, 2003; Yamazaki et al., 2005). On the other hand, *in vivo* experimental models have indicated that the prolonged inhibitory action of CTX promotes the reduction of solid tumors (Faiad, 2012; Brigatte et al., 2016) and ascites tumor (Nunes et al., 2010; Neves et al., 2019). The significance of CTX immunomodulatory activity on macrophages in the tumor microenvironment has been demonstrated as the most marked inhibitory effect on solid tumor development and progression (de Araujo Pimenta et al., 2019). The inhibitory effect of CTX on neovascularization induced by the tumor could significantly contribute to the antitumor action described for this toxin. The present study is the first to demonstrate the direct action of CTX on endothelial cell functions with specificities of the tumor microenvironment, confirming the notable contribution of the anti-angiogenic action to the antitumor effect of this toxin.

## Materials and Methods

### Isolation of Crotoxin

Purification and phospholipase activity of the CTX was performed as described in previous studies (Fraenkel-Conrat and Singer, 1956; Laemmli, 1970; Lobo de Araujo and Radvanyi, 1987; Faure and Bon, 1988; Sampaio et al., 2003; Sampaio et al., 2006a; Sampaio et al., 2006b; Brigatte et al., 2016; de Araujo Pimenta et al., 2019). Briefly, crude venom solution (CdtV) was subjected to anion-exchange chromatography, using a Mono-Q HR 5/5 column in an FPLC system (Pharmacia, Uppsala, Sweden). The fractions (1 ml/min) were eluted using a linear gradient of NaCl (0–1 mol/L in 50 mmol/L Tris-HCl, pH 7.0). Three peaks (p1, p2 and p3) were obtained: p2 corresponded to the pure CTX fraction (about 60% of the crude venom); peaks 1 and 3 included the other CdtV toxins. Before pooling, the fractions containing CTX were tested for homogeneity by non-reducing sodium dodecyl sulphate-polyacrylamide gel electrophoresis (12.5%) and the phospholipase A2 activity was assessed by a colorimetric assay using a synthetic chromogenic substrate.

### Cell Culture

Murine endothelial cells derived from thymus hemangioma (t.End.1) were obtained by courtesy of Dr. Ana Maria Moura (Laboratory of Immunopathology, Butantan Institute, Brazil). The cell line t.End.1 was derived from a thymic hemangioma expressing the polyoma middle T antigen, which is highly tumorigenic and bears functional characteristics similar to those found in angiogenic endothelial cells (Aurrand-Lions et al., 2004; Bussolino et al., 1991; Williams et al., 1988). Cells were cultivated in 75 cm^2^ flasks in the presence of RPMI 1640 (Gibco, Grand Island, NY, United States) media supplemented with 10% fetal bovine serum (FBS), 2 mM L-glutamine, 100 U/mL penicillin, 100 U/mL streptomycin (all Gibco) having a pH 7.4, incubated at 37°C enriched with 5% CO_2_. In all experiments, t.End.1 cells were used between the second and fourth cell passage.

Human breast adenocarcinoma cell line MCF-7 (ATCC^®^ HTB22) were seeded at a density of 1 × 10^6^ in 75 cm^2^ flasks in the presence of RPMI 1640 (Gibco, Grand Island, NY, United States) media supplemented with 10% FBS, 2 mM L-glutamine, 100 U/mL penicillin, 100 U/mL streptomycin (all Gibco), at a pH 7.4, incubated at 37°C, enriched with 5% CO_2_ for growth and semiconfluence. Cells were rinsed twice in phosphate-buffered saline (PBS) and incubated in RPMI 1640 medium containing 2% FBS. Three days later, MCF-7 conditioned medium (MCF-7-CM) was collected, centrifuged for 10 min at 1200 rpm, filtered through a 0.22 µm pore size filter and stored at –20°C until use. In all experiments, MCF-7 cells were used in the second or third cell passage.

### Cell Proliferation and Viability

t.End.1 cells (5 × 10^4^/ml/well) were seeded into a six-well plate in the presence of RPMI 1640 medium and 10% FBS and left overnight at 37°C. Unattached cells were removed after washing with PBS, attached cells were treated with CTX at concentrations of 6.25, 12.5, 25, 50, 100, and 200 nM (corresponding to 0.15, 0.3, 0.6, 1.2, 2.4, and 4.8 µg/ml, respectively), for 24 or 1 h followed by 24 h incubation in fresh culture medium. The cells were then removed from the plate using 0.25% trypsin-EDTA. Cell viability was measured by the dye exclusion method using Trypan Blue. A Neubauer chamber was utilized to determine cell proliferation by cell counting.

### Cell Adhesion Assay

96-well plates were coated with fibronectin (3 µg/ml), type I collagen (10 µg/ml), and laminin (10 µg/ml; Invitrogen, Carlsbad, CA, United States) and left overnight at 4°C. Wells were then washed thrice with PBS and blocked with 1% bovine serum albumin (BSA) in PBS for 2 h at 37°C. For all matrix components evaluated, a negative control (adhesion cells in BSA coating alone) was used to assess adherence to non-specific substrates. t.End.1 cells previously treated with CTX (50 nM) for 1 h in a single cell suspension (1 × 10^6^/ml) were added to the wells and allowed to adhere to the substrate for 1 h at 37°C with 5% CO_2_. After incubation, unattached cells were eliminated by rinsing the well with PBS, while the attached cells were incubated with 5 mg/ml MTT for 3 h at 37°C. Formazan crystals obtained by MTT reduction were dissolved by the addition of 100 µl PBS containing 10% SDS and 0.01 M HCl (18 h, 37°C, 5% CO_2_). The absorbance was read at 595 nm in an ELISA plate reader (Multiskan EX, LabSystem). The number of cells was estimated using the absorbance of a standard curve of known number of fresh live cells added to the plates just before staining (Costa et al., 2013).

### Cell Scratch Wound Healing Assay

A confluent monolayer of t.End.1 (1 × 10^6^) was formed on the coverslips in 24-well plates previously coated with type I collagen (10 µg/ml), and a wound was made using a sterile cell lifter. Next, t.End.1 cells were incubated for 1 h in the presence of CTX (6.25–100 nM). The cells were then washed with PBS and further incubated in the presence of RPMI media containing 1% FBS. After 6, 12 and 24 h of incubation, migrated endothelial cells were stained with Rosenfeld and photographed and quantified with ImageJ software by measuring the area of the cell that moved beyond the reference line. For counting the migrating cells in the field induced by the probe, the images were inserted into the rules on the left and right edges of the field, based on the image obtained in the T0 coverslip. After insertion of dashed lines, the count of migrating cells in the field was performed (de Araujo Pimenta et al., 2019).

### Chemotaxis in the Transwell Model

Transwell inserts (6.5 mm diameter) with an 8 µm pore (Costar, Cambridge, MA, United States) were used to assess the *in vitro* directional migration of t.End.1 cells in response to a gradient of soluble chemoattractants. The membranes were hydrated with serum-free culture media for 45 min at 37°C, containing 5% CO_2_. Next, t.End.1 cells (1 × 10^5^) previously treated with CTX (50 nM) for 1 h were added to the upper side of the inserts in 200 µl serum-free media. The lower chamber was filled with 600 µl of RPMI media supplemented with 2% FBS or MCF-7-CM, which holds high secretory activity for various substances, including chemokines (Garrido et al., 1995; Shih et al., 2012). After 5 h of cell migration at 37°C with 5% CO_2_, non-migrated cells were removed from the upper side of the membrane by cotton swabs. Cells migrated to the lower side were fixed with methanol for 15 min and stained with 0.5% crystal violet for 15 min. After several washing steps with PBS to remove excess amounts of crystal violet dye, cell migration was quantified, and cells were counted from photographs taken under phase contrast microscope in five random fields per insert.

### Capillary-Like Structure Formation Assay on 3D-Matrigel

According to the method described by Arnaoutova and Kleinman (2010), 50 µl of Matrigel (9.3 mg/ml; BD Biosciences, New Bedford, MA, United States) was added to a 96-well culture plate and allowed to polymerize for 45 min at 37°C. Subsequently, t.End.1 cells previously treated with CTX (50 nM) were plated on top of the Matrigel at a density of 1.5 × 10^4^/well in the RPMI medium comprising 2% FBS and incubated for 6 h at 37°C. Tube formation was observed through an inverted phase-contrast microscope (Nikon Eclipse TS100) and photographed with a DS-Fi2 camera, using the Nis-Element D software. Quantification was carried out by counting all branches in five random fields from each well.

### Cell Migration Assays by Time-Lapse Video Microscopy

TTP^®^ 24-well plates were coated with type I collagen (10 µg/100 µl) for 30 min at 37°C. The plates were then washed thrice using PBS, and 1 × 10^3^ cells/well were plated and incubated in RPMI media for 24 h. Next, cells were incubated in the absence (control) or presence of CTX (50 nM) for 1 h. Subsequently, the cells were washed and incubated only in the presence of a fresh culture medium. The plates were incubated and coupled to the IN Cell Analyzer GE 2200 equipment in a ×10 air objective lens; for at least 18 h, eight fields/well (six wells for every treatment) were recorded every 5 min to evaluate t.End.1 cell speed, relative distance, and directionality. Image acquisition was performed using Analyzer 2200, version 1.6.3. The ImageJ plugin manual tracking was employed to track cell nuclei. Hence, the velocity of the cells was analyzed; the net distances per hour were calculated and summed up to determine the total cell path length (Hauff et al., 2015; de Araujo Pimenta et al., 2019). Since the trajectory of individual cells was monitored, directionality was evaluated by calculating the D/T ratio (0–1), which is the ratio between the smallest distance between the initial and final position of the cell (D) by the total distance traveled (T). The effectiveness of migration is improved when directionality is high.

### Confocal Microscopy Analysis of Integrins Distribution and Actin Cytoskeleton Arrangement

Coverslips, previously coated with fibronectin (3 µg/ml), type I collagen (10 µg/ml), and laminin (10 µg/ml) were plated by CTX treated t.End.1 cells (5 × 10^4^/well) and incubated overnight at 37°C with 5% CO_2_ in the presence of MCF-7-CM or RPMI media supplemented with 10% FBS. Subsequently, the cells were fixed in 4% paraformaldehyde and 5% sucrose in PBS buffer for 10 min, rehydrated with PBS (3 × 10 min) and then permeabilized with 0.2% Triton X-100 for 10 min. Unspecific binding sites were blocked with 2% BSA diluted in PBS for 1 h at room temperature (RT). After that, t.End.1 cells were incubated with primary rabbit antibodies against αv integrin for fibronectin coating and α2 integrin for type I collagen coating (both antibodies from Millipore, Billerica, MA, United States) for 1 h at RT. After washing, secondary antibodies (Goat anti-rabbit Alexa 488, 1:800; Invitrogen, Carlsbad, CA, United States) and rhodamine phalloidin (1:800; Molecular Probes, Burlington, CA, United States) were applied for 1 h at RT. Negative controls consisted of the absence of primary antibodies. The coverslips were washed twice in PBS, mounted with Vectashield^®^ (Vector Labs, Burlingame, CA, United States) and observed using a Zeiss confocal inverted microscope (Zeiss LSM-510) (Butantan Institute, São Paulo, Brazil).

### SDS/PAGE and Western Blot Analysis

The t.End.1 cells previously treated with 50 nM of CTX for 1 h were harvest and lysed in Radioimmunoprecipitation assay (RIPA) buffer (R0278; Thermo Scientific) containing 1:300 protease and phosphatase inhibitor cocktail (P8340, P5726, P0044; Sigma-Aldrich) and incubated on ice for 30 min. Cell lysates were homogenized and then centrifuged at 16,000 × g at 4°C for 20 min. The supernatant was collected and protein concentration was measured using BCA Protein Assay. Protein extracts (30 µg) were denatured in Laemmli buffer, incubated at 95°C for 4 min and then, were separated into 4–20% polyacrylamide gels (Bio-Rad). After electrophoresis, samples were transferred to a nitrocellulose membrane (Bio-Rad). The membranes were blocked in Tris Buffered Saline with 0.1% Tween^®^ 20 (TBST) containing 5% BSA for 2 h at RT and then incubated with anti-FAK antibody (1:1000; BD Biosciences), anti-Arp2/3 (1:1000; Abcam), anti-F-actin (1:500; Abcam), anti-Rac1 (1:1000; Abcam), anti-MMP-2 (1:2000; Millipore) and anti-MMP-9 (1:2000; Millipore) overnight at 4°C The membranes were then incubated in the peroxidase-conjugated secondary antibody (1:5000; anti-rabbit or anti-mouse) for 2 h at RT. Enhanced Chemoluminescence kit (Thermo Scientific) was used for detection. The signals were detected using an image acquisition system (Uvitec mod Alliance 9.7; Uvitec, Cambridge, United Kingdom). Band intensities were measured using ImageJ (NIH) software. Targeted bands were normalized to the GAPDH antibody (1:5000; Abcam).

### Measurement of VEGF, MMP-2 and Pro-MMP-9

Endothelial cells were treated with 50 nM CTX and then incubated in RPMI or MCF-7 tumor-conditioned media for 24 h. The supernatant was collected, and the concentration of the VEGF, MMP-2 and pro-MMP-9 thus secreted, was measured using an enzyme-linked immunosorbent assay (ELISA, Abcam Elisa Kit).

### Statistical Analysis

GraphPad InStat software version 3.01 (GraphPad Software Inc., San Diego, CA, United States) was used (Glantz, 1997) for the statistical analyses. Multiple comparisons analyses (for all pairs of groups) were performed using one-way analysis of variance (ANOVA) followed by Tukey’s post test. Results from other assays were analyzed using ANOVA and then Bonferroni’s test for multiple comparisons against a single control. An unpaired Student’s t-test or Mann Whitney test was performed to compare two groups. A *p*-value <0.05 was considered statistically significant. The results have been demonstrated as mean value ± standard errors of means.

## Results

### CTX Inhibits Proliferation and Adhesion of t.End.1 Cells to Different Extracellular Matrix Ligands

**FIGURE 1:**
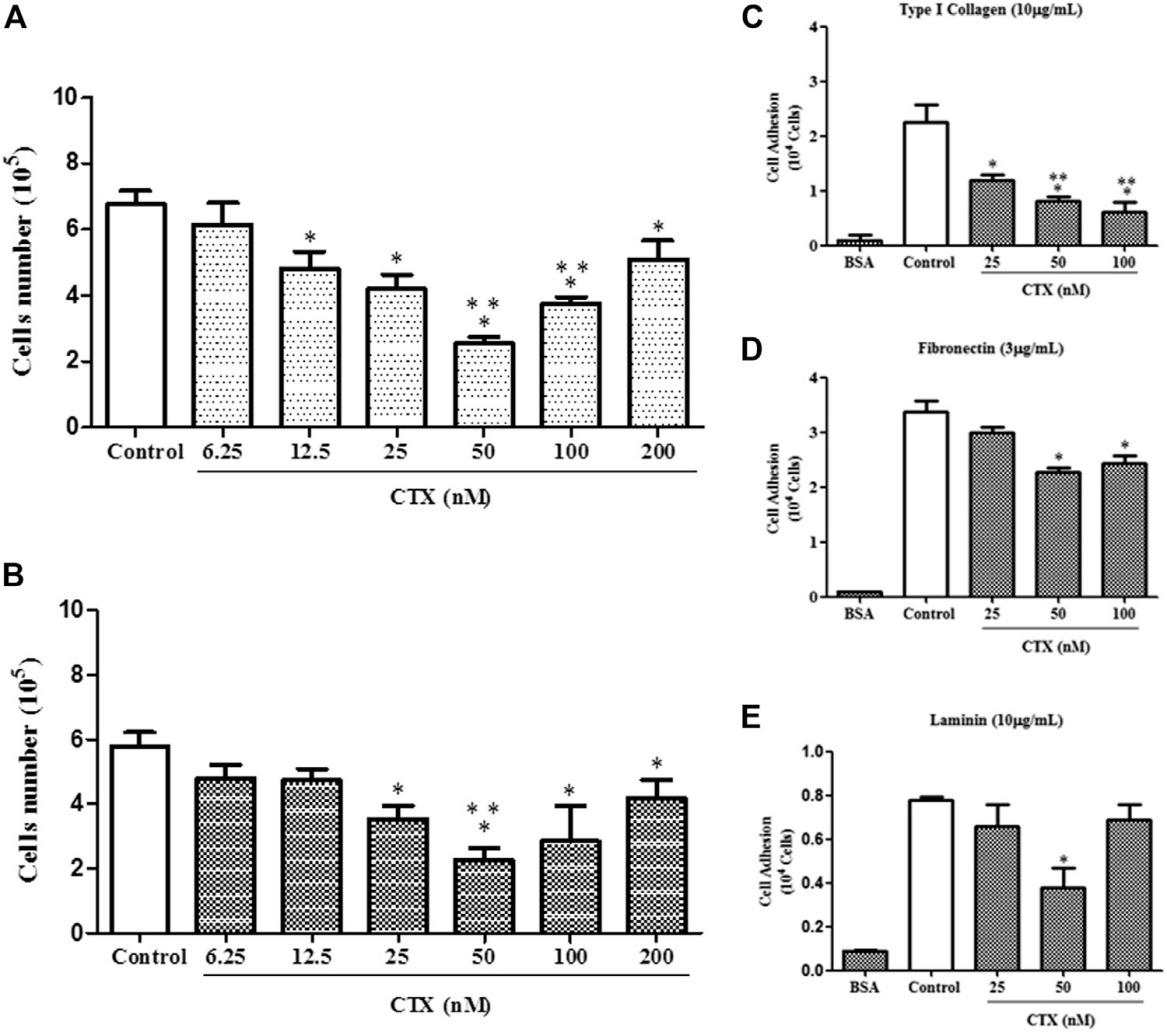
Effect of CTX on t.End.1 cell proliferation and adhesion to extracellular matrix ligands. t.End.1 cells (5 × 10^4^ cells/well) were incubated in the presence of different CTX concentrations (6.25, 12.5, 25, 50, 100 and 200 nM) for 24 h **(A)** or were previously incubated with the different concentrations for only 1 h and then washed and incubated for another 24 h only in fresh culture medium **(B)**. Cell proliferation was assessed after 24 h by cell counting. The data are presented from three distinct experiments run in octoplicate and are expressed as mean ± s.e.m. *p < 0.05 compared to control group, **p < 0.05 compared to other CTX concentrations. For adhesion assay, t.End.1 cells pretreated for 1 h with CTX (25, 50 and 100 nM) were washed and added (100 µl) to Maxsorp plates (Nunc^®^) containing 96 wells, previously sensitized with the different ligands of matrix: type I collagen (10 µg/ml) **(C)**; fibronectin (3 µg/ml) **(D)** and laminin (10 µg/ml) **(E)**. After 1 h, adhered cells were evaluated by MTT assay. The values obtained were fed into GraphPad INSTAT program V2.01 for conversion of optical density (OD) in the number of adhered cells. The data are presented from three distinct experiments run in sextuplicate and are expressed as mean ± s.e.m. *p < 0.05, compared to control group and **p < 0.01, significantly different from mean values for groups to their respective CTX-treated cells.

Initially, the proliferative capacity of t.End.1 cells were evaluated 24 h after incubation in the presence of CTX at different concentrations. A significant inhibition of endothelial cell growth was observed at concentrations of 12.5 nM (29%), 25 nM (38%), 50 nM (62%), 100 nM (44%), and 200 nM (24%), compared to the control group, consisting of t.End-1 cells incubated only in the presence of RPMI 1640 medium, under the same experimental conditions. Only the 6.25 nM concentration did not affect the number of cells (Figure 1A). To evaluate whether short incubation in the presence of CTX would have the same inhibitory effect as observed in long incubation, t.End.1 cells were pretreated with CTX for 1 h at the same concentrations and subsequently washed and incubated only in the presence of fresh culture medium for 24 h. The results demonstrated that CTX significantly inhibited the proliferative capacity of t.End.1 cells at concentrations of 25 nM (39%), 50 nM (61%), 100 nM (51%), and 200 nM (27%) compared to the control group, consisting of t.End-1cells incubated only in the presence of RPMI 1640 medium, under the same experimental conditions. CTX concentrations of 6.25 and 12.5 nM showed inhibition of 17 and 18%, respectively, but were not considered statistically significant in comparison to the control group, incubated only in the presence of RPMI culture medium (Figure 1B). Cell viability test (1% Trypan blue exclusion) was performed after the assays, both on cells incubated with culture medium (control) and CTX. The viability of all cells was more than 98% (data not shown). Based on these results, for the accomplishment of other assays t.End.1 cells were treated with CTX beforehand for 1 h.

To investigate the action of CTX on t.End.1 cell adhesion on different natural ligands, main concentrations of the toxin that induced inhibition on the proliferation of t.End.1 cells were utilized. CTX at concentrations of 25, 50, and 100 nM inhibited (47, 64, and 72%, respectively) cell adhesion to type I collagen (10 µg/ml) significantly as compared to the control group (Figure 1C). On the other hand, endothelial cell adhesion on fibronectin coating (3 µg/ml) was significantly affected at CTX concentrations of 50 nM (33%) and 100 nM (28%), in comparison to the control group (Figure 1D). Unlike other matrix components, t.End.1 cells showed a lesser adhesion to laminin (10 µg/ml) coating (Figure 1E). Furthermore, the inhibitory effect of CTX was observed only at a concentration of 50 nM (55%), relative to the control. Negative control (BSA coating) demonstrated that cell adhesion was ECM protein-dependent (Figures 1C–E).

### CTX Decreases Migration in Wound Healing Model

**FIGURE 2:**
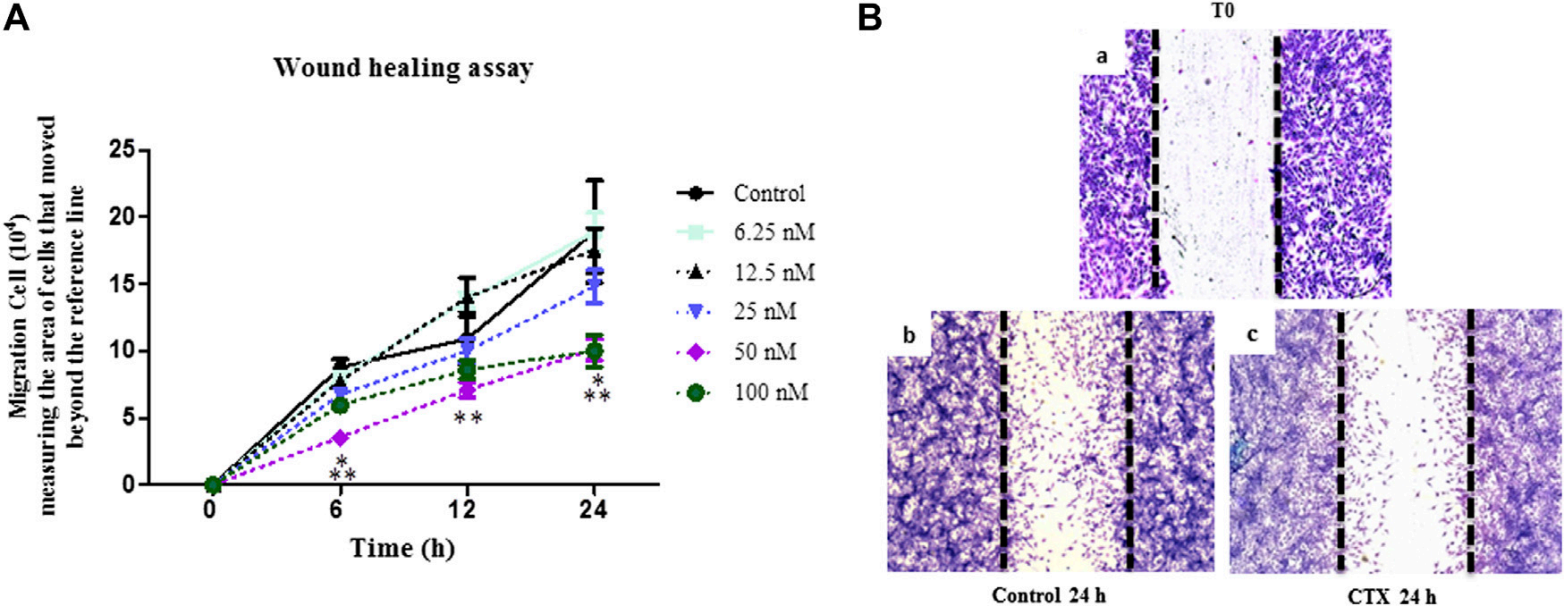
Effect of CTX on t.End.1 migration in the Wound healing model at different treatment periods. Wound healing assay performed in t.End.1 cells treated with different CTX concentrations (6.25, 12.5, 25, 50, 100 and 200 nM) for 0, 6, 12 and 24 h at 37°C and 5% CO_2_. **(A)** Results are expressed as Number of Migrated Cells and represent the mean ± s.e.m. The data are presented from three distinct experiments run in at least in triplicate each group. *p < 0.05 compared to the control group, **p < 0.05 compared to other CTX concentrations. **(B)** Photomicrographs obtained at time 0 and 24 h at ×10 magnification. The images were collected under an Olympus BX 51 microscope, using the Image-Pro Plus 5.1 software, in a ×10 objective.

Wound healing assay was used to evaluate the directional endothelial cell movement onto an empty field created by an interruption in the cell monolayer. After that, t.End.1 cells were treated with different concentrations of CTX (6.25–100 nM) for 1 h. Subsequently, cells were incubated in culture medium for 6, 12, and 24 h. The significant inhibitory action of CTX on t.End.1 cell migration was observed at concentrations of 50 and 100 nM (59 and 33%, respectively) after 6 h, as compared to the control cells (Figure 2A). As compared to other concentrations (6.25 nM: 55%, 25 nM: 54%, 50 nM: 46%, and 100 nM: 39%), CTX significantly inhibited cell migration at a concentration of 50 nM within 6 h (Figure 2A). In the 12 h period, the inhibitory action of CTX at concentrations of 50 and 100 nM was again significant, in comparison to the control cells (35 and 21%, respectively) and with the other concentrations (6.25 nM: 49 and 38%, respectively and 12.5 nM: 49 and 39%, respectively) (Figure 2A). Following 24 h of incubation, cell migration was significantly inhibited when pretreated at concentrations of 50 and 100 nM (47%), in comparison to the control cells, and the concentration of 6.25 nM (Figure 2A). Figure 2B depicts the results obtained over the 24 h culture period, after 1 h pretreatment with 50 nM CTX. The time 0 h corresponds to the period in which the monolayer was interrupted. After 24 h, inhibited migration of t.End.1 cells into the empty field was observed following pretreatment with CTX.

### CTX Prevents Capillary Structure Formation by t.End.1 Cells Grown in 3D-Matrigel and Compromises the t.End.1-Migratory Behavior on Type I Collagen in a 2D Assay Analyzed by Time-Lapse Method

**FIGURE 3:**
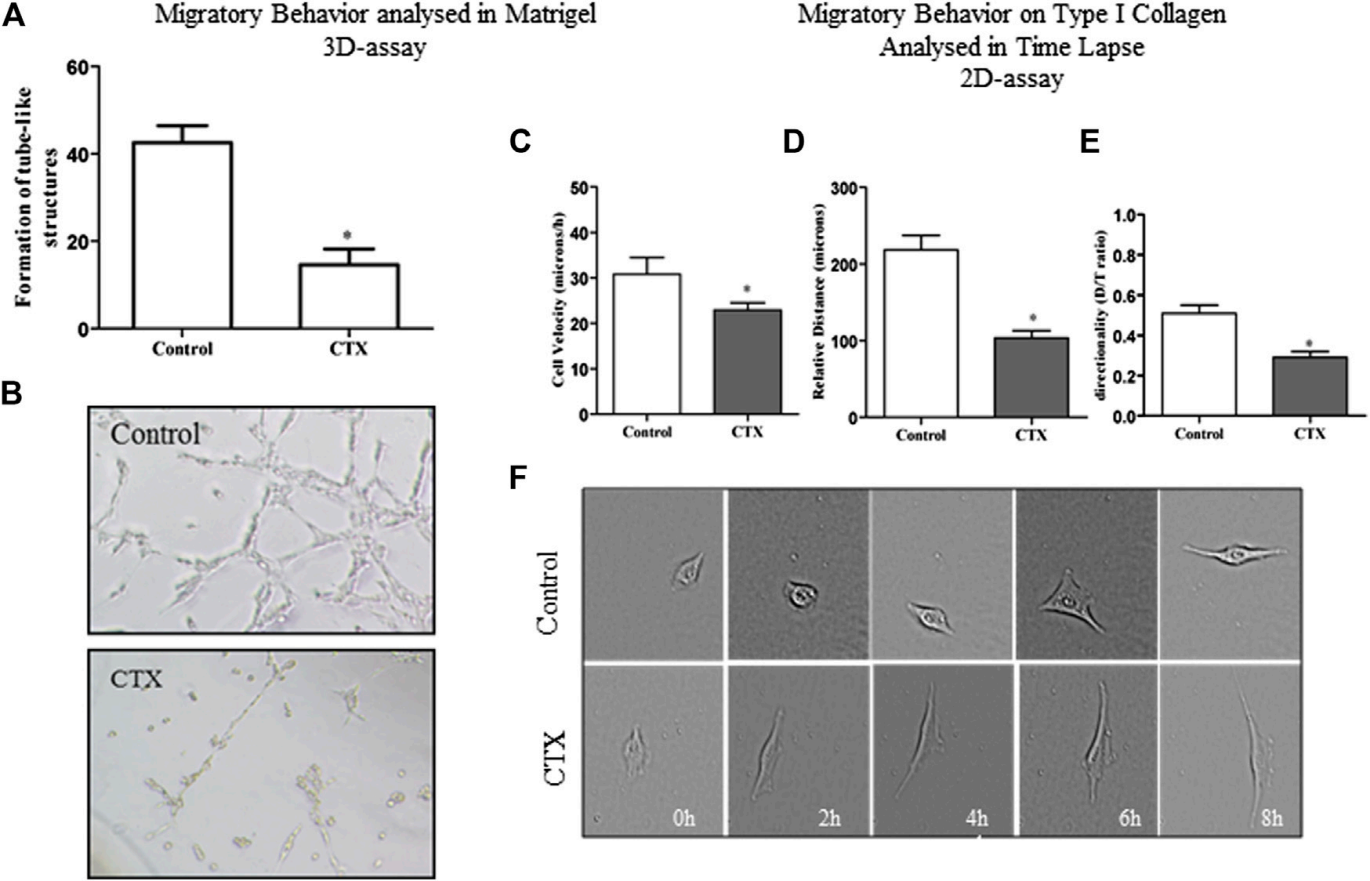
Effect of CTX on the formation of tubule-like structures in 3D-matrigel and migratory behavior on type 2D-type I collagen evaluated in Time Lapse. CTX-pretreated t.End.1 cells were added on polymerized Matrigel in 96-well plates and incubated for 6 h. The results are expressed in a number of tubular structures and represent the count of five fields from triplicates of each group. The data are presented from three distinct experiments. *p < 0.0001, compared to the control group **(A)**. Photomicrograph of representative tube formation. Images obtained at the ×40 objective **(B)**. For the 2D-assay, time lapse images obtained from CTX pretreated t.End.1 cells migrating under collagen coating in In Cell Analyzer GE 2200 equipment in a ×10 objective for 18 h. The t.End.1 cell velocity **(C)**, relative distance **(D)**, and directionality **(E)** were measured. These parameters were evaluated using Image J software. Data are mean ± s.e.m. *p < 0.05 by comparison with the control group (*n* = 29) and CTX (*n* = 33). The graphs were plotted from the data collected during three independent experiments.

The effect of CTX on the formation of endothelial cell capillary structures was evaluated in 3D-Matrigel (Figure 3). Control cells showed capillary structure formation after the second hour of incubation (data not shown). After 6 h, the t.End.1 cellular network thus formed presented fine, elongated structures, and cell-cell contact was established (Figures 3A,B, Panel Control). On the other hand, t.End.1 cells pretreated with CTX (50 nM) for 1 h demonstrated a 66% reduction in the ability to form capillary-like structures compared to control cells at the same incubation period. This result suggests that CTX promoted loss of cell-cell contact, possibly accompanied by cytoskeleton retraction, contributing to the reduction of capillary-like structures (Figures 3A,B, panel CTX).

A detailed analysis of the endothelial cell dynamic behavior during migration was analyzed by time-lapse microscopy, which allows measurement of individual parameters such as cell velocity, distance traveled (relative distance), and directionality. A 2D assay was performed where t.End.1 cells at low density were plated on dishes previously coated with a mixture of type I collagen (10 μg/ml) and 1% fibronectin (3 μg/ml). It was found that after 8 h of incubation, CTX reduced 25% of t.End.1 migration velocity at basal stimulus compared to the control group (22.95 ± 1.61 vs. 30.8 ± 3.7) (Figure 3C). Besides, CTX also decreased relative distance (Figure 3D) and directionality (Figure 3E) of t.End.1 cells (53 and 43%, respectively). Figure 3F represents the results obtained in the 8 h culture period, after pretreatment with 50 nM CTX for 1 h, and control cells stimulated with RPMI 1640 medium supplemented with 2% FBS.

The Supplementary Material (Supplementary Videos S1,S2) projects the migratory capacity through time-lapse assay of the t.End.1 cells in a 2D-matrix of type I collagen and fibronectin mixture, cultured in the presence of the RPMI medium supplemented with 2% FBS for 8 h (Supplementary Videos S1,S2). The images depict that t.End.1 cells treated with CTX (Supplementary Video S2) exhibited altered migratory behavior and changed the cytoskeletal dynamics and the formation of lamellipodium-like structures, compromising cytoskeleton displacement and directionality *via* 2D coating.

### CTX Impairs t.End.1 Migration Properties in MCF-7-Conditioned Medium

#### Migration in Wound Healing Model

**FIGURE 4:**
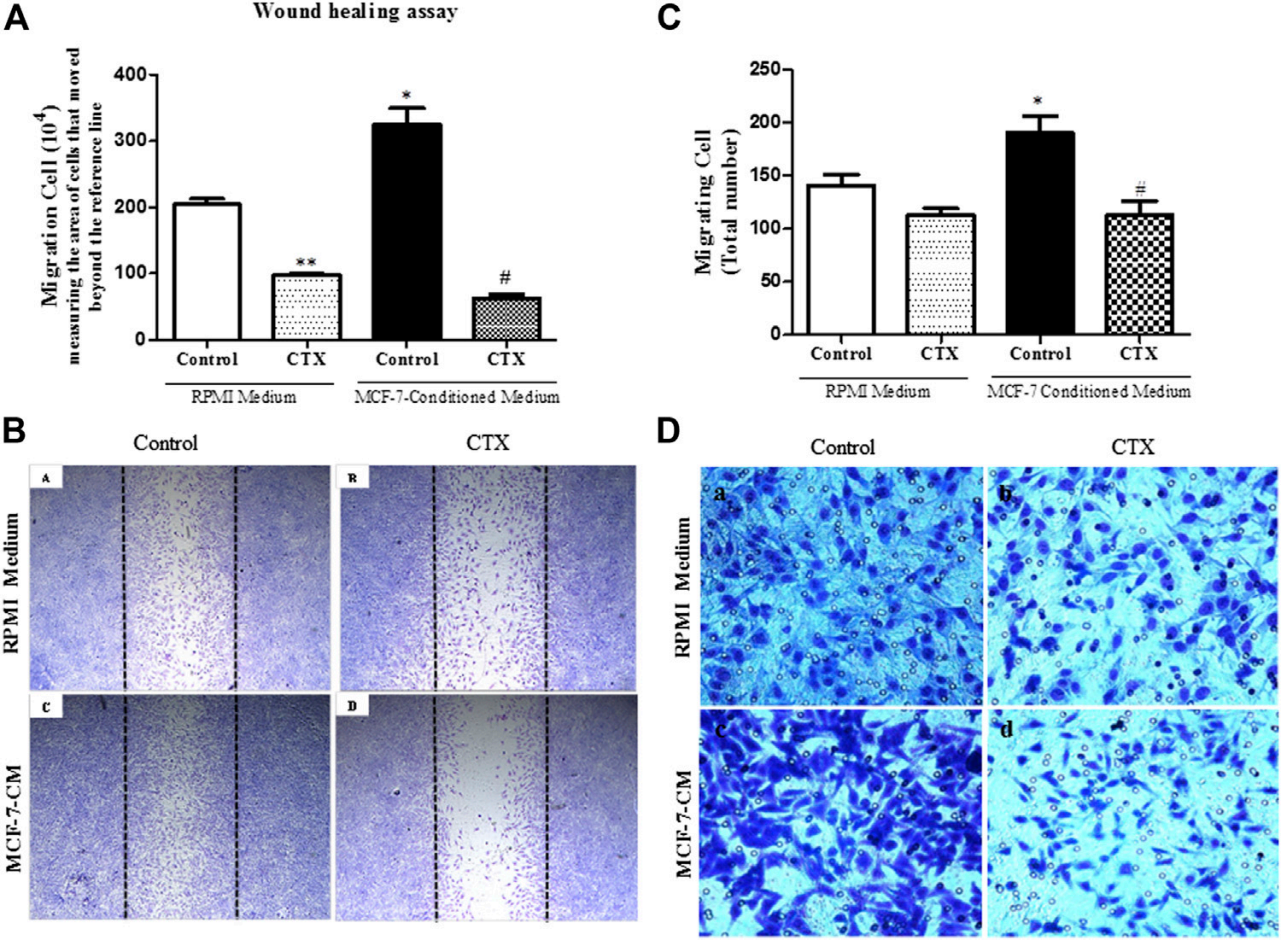
Effect of the CTX on migration assay induced by MCF-7-Conditioned medium. **(A)** Wound healing assay performed in CTX-pretreated t.End.1 cells in the presence of RPMI medium containing 10% FBS (control) or in the presence of MCF-7-conditioned medium (chemotactic stimulation) for a period of 24 h. After this time, cells were fixed by the Rosenfeld panchromic method. A total of five random fields per coverslip were counted under a brightfield microscope (Standard 25; Carl Zeiss, Germany) using a ×10 objective. The results are expressed as Number of Migrated Cells and represent the mean ± s.e.m. The data are presented from three distinct experiments run in quadruplicate of each group. *p < 0.001 compared to RPMI control group. **p < 0.001 compared to RPMI control group. #*p* < 0.001 compared to MCF-7-CM control group. The images represented in **(B)** in Panels **(a**–**d)** were obtained in a ×5 objective. Panel **(a)** represents control cells incubated in RPMI 1640 medium with 10% FBS; Panel **(b)** represents cells treated with CTX and incubated in RPMI 1640 medium with 10% FBS; Panel **(c)** represents control cells incubated in MCF-7-CM and Panel **(d)** represents CTX treated cells and incubated in MCF-7-CM. For the Transwell assay, in **(C)** the number of migrating cells was determined by counting in five random fields per membrane using light microscopy. The results are expressed as Migrant Cells and represent the mean ± s.e.m. *p < 0.001 compared to RPMI control group. #*p* < 0.01 compared to MCF-7-CM control group. **(D)** The panels are representative of t.End.1 cell chemotaxis: **(a)** control in response to RPMI 1640 medium with 2% FBS; **(b)** CTX in response to RPMI 1640 medium with 2% FBS; **(c)** control in response to MCF-7-CM; **(d)** CTX in response to conditioned medium from MCF-7 tumor cells. The images were collected under an Olympus BX 51 microscope using the Image-Pro Plus 5.1 program, using a ×40 objective. The data are presented from three distinct experiments.

Under basal stimulus (RPMI supplemented with 2% FBS), t.End.1 cells previously treated with CTX (50 nM) and incubated for 24 h demonstrated a significant reduction (52%) in endothelial cell migration (Figures 4A,B, a,b [if subparts are of B]). However, under tumor-derived factors stimulus by the use of tumor conditioned medium at the same experimental conditions, a 59% increase in the migratory capacity of t.End.1 cells was demonstrated compared to the basal stimulus group (Figures 4A,B, a,c [if subparts are of B]). Surprisingly, t.End.1 cells pretreated with CTX and subjected to the migration assay under tumor conditioned medium stimulus demonstrated a marked inhibitory effect (81%) compared to that in the control cells pretreated with RPMI supplemented with 2% FBS alone (Figures 4A,B, c,d [if subparts are of B]).

#### Migration in Transwell Model

As illustrated in Figure 4C, the control group demonstrated an increased cell migration (35%) under conditioned media with MCF-7 stimulus, in comparison to the control group stimulated with RPMI media having 2% FBS (Figures 4C,D, a,c [if subparts are of D]). Furthermore, t.End.1 cells treated with CTX (50 nM) for 1 h supported 41% inhibition of cell migration against the control group stimulated with MCF-7 conditioned medium (Figures 4C,D, c,d [if subparts are of D]). Regarding basal stimulus, although CTX showed 21% inhibition of the number of migrated cells, this difference was not statistically significant when compared to the control group (Figures 4C,D, a,b [if subparts are of D]).

### CTX Inhibits MMP-2 and VEGF Secretion and MMP-2 and MMP-9 Expression

**FIGURE 5:**
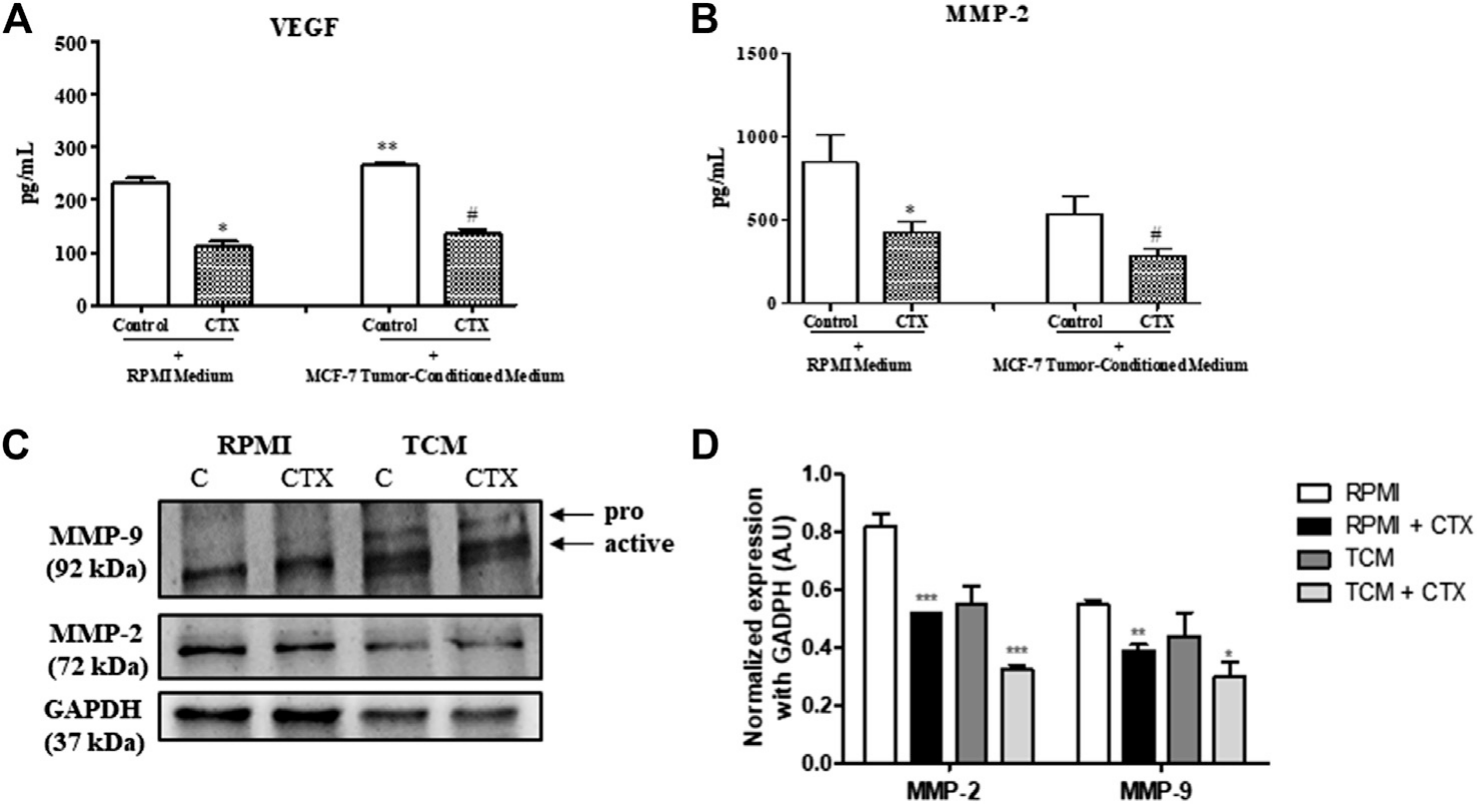
Effect of CTX on the VEGF and MMPs. CTX-pretreated t.End.1 cells were incubated for 24 h in the presence of the fresh medium. Then, the supernatants were collected 99 for measurement of VEGF **(A)** and MMP-2 **(B)** concentrations by means of enzyme immunoassay (EIA) using a commercial kit. Data are mean ± s.e.m. Data are the average of four samples of each group ± s.e.m and represent two distinct trials. *p < 0.01, compared to RPMI control group. **p < 0.05, compared to RPMI control group, #*p* < 0.01, compared to MCF-7-CM. Western blotting analysis of protein expression levels of MMP-2 and MMP-9 **(C)**. The values were normalized by GAPDH expression and band intensities were quantified by densitometry of the homogenate Western blots **(D)** and represented the mean ± s.e.m. for three samples per group and represent three independent assays. *p < 0.05 by comparison with the respective control groups. **p < 0.01 by comparison with the respective control groups. ***p < 0.001 by comparison with the respective control groups.

CTX significantly inhibited the concentration of VEGF in t.End.1 cells in the presence of basal media and tumor conditioned media compared to the control (52 and 49%, respectively) (Figure 5A). The MMP-2 and MMP-9, which are related to the degradation of the basement membrane in the process of angiogenesis, demonstrated that CTX inhibited MMP-2 secretion under both the stimuli compared to its respective control (50 and 47%, respectively) (Figure 5B). Conversely, CTX did not have any effect on pro-MMP-9 secretion (data not shown). Western blotting analysis has shown the effect of CTX on MMP-9 and MMP-2 protein levels. Similar results were found on MMP-2 where CTX also inhibited MMP-2 expression by 37 and 41% under basal and tumor stimuli, respectively. Regarding MMP-9 protein expression, the anti-MMP-9 antibody detected both pro-MMP-9 and active MMP-9 as breakdown products, there is no detection of pro-MMP-9 expression level in the RPMI group and there is a mild expression in the TCM group which can be correlated to the finding of ELISA assay. However, the active MMP-9 expression levels were detected and were inhibited by CTX under both basal and tumoral stimuli (29 and 32%, respectively). (Figures 5C,D).

### CTX Interferes in t.End.1 Filamentous Actin Organization Under Different Stimuli by Inhibiting Proteins Involved in the Signaling Pathway of Different Integrins

**FIGURE 6:**
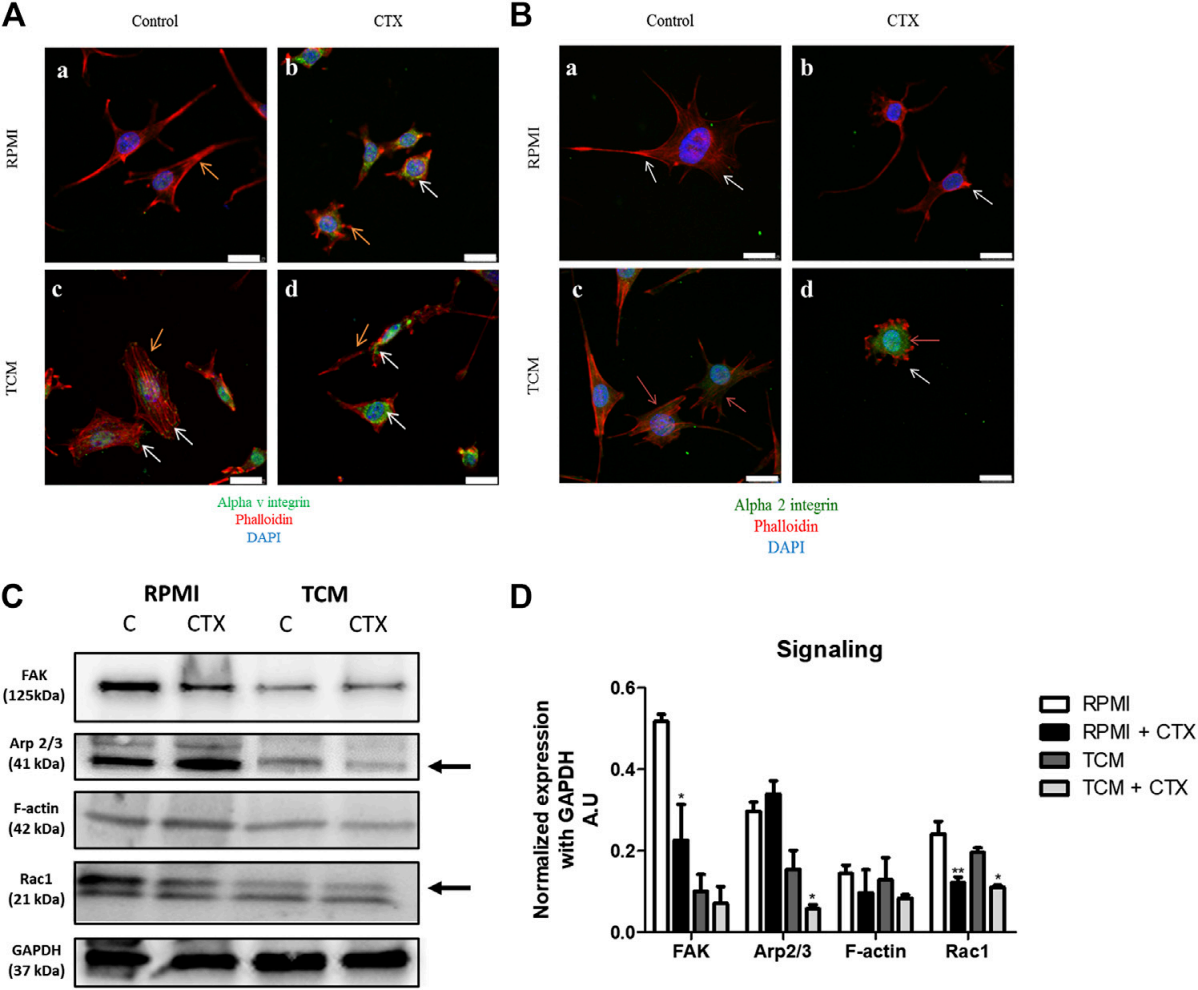
Effect of CTX on actin cytoskeleton, intracellular signaling and integrin expression in t.End.1 endothelial cells in the different microenvironments. In **(A)**, marking of αv subunit (green) and F-actin (red) of control cells incubated with RPMI 1640 medium **(a)** or MCF-7-CM **(b)**. CTX-treated cells are labeled in **(b**,**d)**. In **(B)**, marking of α2 subunit (green) and F-actin (red) of control cells incubated with RPMI 1640 medium **(a)** or MCF-7-CM **(b)**. CTX-treated cells are labeled in **(b**,**d).** Orange arrows represent the expression of αv and α2 in the cell (green); White arrows represent the cytoskeleton protrusions (red). For details of the experiments, please see the Materials and Methods section. Scale bar: 25 μm. Western blotting was performed for the analysis shown in **(C)**, integrin-mediated signaling: FAK, Arp2/3, F-actin and Rac-1 expression levels. The values were normalized by GAPDH expression and band intensities were quantified by densitometry of the homogenate western blots and represented the mean ± s.e.m. for three samples per group and represent three separate assays **(D)**. *p < 0.05 by comparison with the respective control groups. **p < 0.01 by comparison with the respective control groups. ***p < 0.001 by comparison with the respective control groups.

The filamentous actin (F-actin) cytoskeleton of t.End.1 cells, incubated overnight in RPMI medium with 2% FBS on both fibronectin (Figure 6A, a [if subparts are of A]) and type I collagen (Figure 6B, a [if subparts are of B]) showed a distinct cell cortex, a few actomyosin stress fibers (structures responsible for the production and transmission of mechanical stress) and, most notably, multiple projections in different directions (filopodia and lamellipodia). In the presence of MCF-7-CM control cells showed a marked increase of stress fibers (SFs), becoming more contractile; on fibronectin, cells were apparently more polarized, showing a morphology that favors directional migration (Figures 6A,B, c [if subparts are of both A and B]). Treatment with CTX led to F-actin disorganization, dissolution of stress fibers and retraction of cellular projections, especially on fibronectin, under both the experimental conditions (RPMI-1640 and MCF-7-CM) (Figures 6A,B, b,d [if subparts are of B]).

The distribution of αv integrins on control and MCF-7-CM treated cells plated on fibronectin showed their presence in focal adhesions to the extracellular matrix, where stress fibers were inserted (Figure 6A, a,c [if subparts are of A]). Treatment with CTX on both experimental conditions (control and MCF-7-CM), maybe due to its collapsing effect on the F-actin cytoskeleton, led to an intracellular localization of this integrin subunit, with concentration in the perinuclear region (Figure 6A, b,d [if subparts are of A]).

Regarding the signaling proteins, CTX inhibited FAK (58%) and Rac1 (49%) expression with no effects on the levels of Arp2/3 (a Rac1 effector protein) and G-actin expression at basal condition. (Figures 6C,D). The Rac2 and Rac3 proteins have similar molecular weight, both being 21 kDa. Therefore, the other bands observed in the gel may be the product degradation of Rac 1 (Figure 6C). The effects of tumor-conditioned medium on control cells were a quite pronounced reduction on the expression of FAK and Arp 2/3 proteins, with no significant changes in Rac1 and actin filaments (Figures 6C,D). Cells pretreated with CTX and incubated in MCF-7-CM compared to MCF-7-CM control, showed higher inhibition of Arp2/3 (63%); the expression of Rac1, not previously affected by the tumor conditioned medium, was also reduced by CTX (43%) (Figures 6C,D). Science Identifiers (LSIDs) for ZOOBANK registered names or nomenclatural acts should be listed in the manuscript before the keywords with the following format:

## Discussion

The study demonstrated that CTX interferes with the functions of endothelial cells involved in tumor angiogenesis. The evaluation of whether this toxin could alter angiogenic events like the proliferation, adhesion, and migration activities of t.End.1 cells revealed that the presence of CTX affected the proliferative capacity of endothelial cells. The inhibitory effect was mainly observed at intermediate concentrations (25, 50, and 100 nM). These results have already been observed in other experimental models, where the median concentrations of the curve were found to be the most potent (Faiad et al., 2008). However, the inhibitory activity is probably not due to any cytotoxic effect of CTX on endothelial cells.

Cell proliferation depends on the degree of adhesion between the cell and its extracellular matrix ligand. It was noted that t.End.1 cells showed good adhesion to type I collagen and fibronectin, with values similar to those reported previously in the literature (Schlie-Wolter et al., 2013), and unlike that observed with these matrix components, the adhesiveness of t.End.1 was lower to laminin. Cell adhesion depends on the isoform of both integrin and laminin for specificity and affinity (Nishiuchi et al., 2006). Cell migration is an essential part of tumor progression. All CTX concentrations were assessed for endothelial cell migration. As the study was conducted in a wound healing model, it was possible to observe the inhibitory capacity of CTX concentrations, particularly the 50 nM concentration, on cell migration induced in type I collagen coating. The results obtained after cell proliferation and migration tests in the wound healing model defined the use of 50 nM concentration for other experimental assays. Contributing to this negative effect on cell migration, CTX reduced cellular adhesion to collagen type I, fibronectin and laminin. Lowered expression of important integrins and cytoskeletal changes were related to reduced migration, with consequences to the ability of endothelial cells to form new vessels.

The capillary-like structure formation is an essential step in the angiogenesis process. Matrigel, a reconstituted basement membrane, was used to study *in vitro* angiogenesis as it allows rapid formation of capillary-like structures in a 3D system, being a simple and easily quantifiable technique (Arnaoutova et al., 2009; Bazaa et al., 2010). The presence of CTX also affected the formation of capillary-like structures. The toxin CTX altered endothelial cell morphology by preventing its projection to Matrigel. It can be suggested that Matrigel, derived from EHS murine sarcoma, provides conditions like those in the tumor microenvironment, allowing the rapid formation of tubular structures, as observed in this study. The results of this assay corroborate the data obtained in the adhesion and cell migration assays, which proposes that CTX affects the significant processes that depend directly on cytoskeleton reorganization.

The actin cytoskeleton is directly associated with the adhesive and migratory behavior of endothelial cells anchored to integrins that are adhesion molecules with bidirectional communication. Integrins allow the interaction between cells and extracellular matrix components (Weis and Cheresh, 2011), support the actin cytoskeleton’s reorganization, and consequently, the cell migration, thus thriving the angiogenic process. Pretreatment with CTX reduced velocity and persistence in cells migrating on collagen type I, resulting in smaller distances traveled, corroborating the results observed using the wound healing assay. CTX also disorganized the actin cytoskeleton, and cells showed less stress fibers, shorter filopodia and smaller lamellipodia – all cellular processes important for cell migration.

The inhibitory action of CTX on the tumor stimulus was assessed by performing a migration assay using two *in vitro* models: wound healing and transwell system for chemotaxis. In the Wound healing test, migration is directional (stimulated by the simulated wound), compared with the transwell model, which is directional migration stimulated by chemotaxis. At basal condition (in the presence of RPMI 1640 medium supplemented with 2% FBS), CTX at concentrations of 50 and 100 nM significantly inhibited cell migration from the 6thh onwards, 12thh, and the 24thh. Twenty-four hours is the maximum period analyzed in the wound healing model in all studies to date (Denker and Barber, 2002; Hotchkiss et al., 2002; Durham and Herman, 2009). The concentration of 50 nM caused the most remarkable inhibitory action among all. CTX entails its most striking actions at median doses in different cells (Faiad et al., 2008; Faiad et al., 2008; Lima et al., 2012; Costa et al., 2013). Thus, 50 nM concentration was used for the mediated supernatant wound healing assay obtained from tumor cells. At this concentration, CTX significantly inhibited cell migration induced by the MCF-7-CM. Similar results were obtained in the chemotaxis assay, demonstrating that CTX, at the concentration used, significantly inhibits the migration of endothelial cells against tumor stimulus. However, when basal conditions were used as a stimulus, no significant inhibition was observed. Notably, the conditioned medium used in the chemotactic factor migration assay was derived from a highly invasive human breast tumor line (MCF-7) owing to its ability to secrete various growth factors, including chemokines (Uddin et al., 2018), which are pro-inflammatory and modulate the angiogenic process (Romagnani et al., 2004).

Once the inhibitory action of CTX on functional events, like adhesion, proliferation, and migration of endothelial cells, including the tumor microenvironment was estimated, the mechanisms involved in this inhibitory activity were analyzed. Angiogenic functions of the endothelial cell involve the participation of integrins and their signaling pathways, coordinating both, the functions linked to the cytoskeleton, and the production and secretion of mediators (Bianconi et al., 2016; Guerrero and McCarty, 2018).

Accordingly, the concentrations of MMPs (MMP-2 and MMP-9) and VEGF were measured from the t.End.1 endothelial cell supernatants irrespective of whether they were pretreated with CTX (50 nM). It was revealed that CTX significantly reduced MMP-2 and VEGF secretion. An association between the MMP-2 and αvβ3 integrin was identified, suggesting the co-localization between these proteins on the membrane surface of endothelial cells (van Hinsbergh et al., 2006). Alternatively, this integrin captures latent MMP-2 to initiate extracellular matrix degradation (Brooks et al., 1996). Hence, it is proposed that the inhibitory action of CTX on αv subunit expression and distribution may contribute to the decreased secretion of this metalloproteinase by endothelial cells. The same inhibitory potential of CTX on MMP-2 secretion was observed against tumor stimulation. Although different studies have revealed the significance of MMP2 and MMP-9 in angiogenesis, mainly related to the tumor, the inhibitory action of CTX on pro-MMP-9 secretion was not observed in this study (data not shown). On the other hand, the expression levels of MMP-2 and active MMP-9 detected by western blotting demonstrated the significant inhibitory action of CTX and thus, reinforcing the capacity of this toxin in inhibiting crucial angiogenic-mediators.

As reported in the literature, αvβ3 integrin (RGD-recognizing integrins) can bind to different matrix components, including fibronectin and collagen I; this integrin is directly related to tumor angiogenesis (Hood and Cheresh, 2002; Bazaa et al., 2010). The integrin αvβ3 is expressed on the surface of endothelial cells (Aurrand-Lions et al., 2004; Davis and Senger, 2005). The main integrins responsible for the interaction between endothelial cell and collagen are α1β1 (preferentially recognizing type IV collagen) and α2β1 integrins, which mainly bind to type I collagen (Arlinghaus et al., 2013; Guerrero and McCarty, 2018) and laminin. After adhesion, these integrins alter the shape of these cells to promote cell migration (Languino et al., 1989; Davis and Senger, 2005; Guerrero and McCarty, 2018).

The immunofluorescence assay verified that CTX-treated endothelial cells altered the distribution of αv (fibronectin coating) and α2 (type I collagen and laminin coating) integrin subunits, in comparison to the control. Consequently, this alteration may have triggered changes in the actin cytoskeleton, promoting disruption in cell projection under laminin coating, disorganization, and reduction of SFs under collagen and fibronectin coating, respectively, compromising cytoskeleton contraction. Besides, punctual actin accumulation was observed in collagen and fibronectin coating. So, these actin cytoskeleton modulations and protrusions, promoted by the presence of CTX, may have been due to decreased migration-related intracellular signaling (Ridley et al., 2003) and possibly, decreased expression of integrin subunits. Consequently, low adhesion between endothelial cells and the matrix component prevented the generation of sufficient tensile force for efficient migration (Hood and Cheresh, 2002). In conditions stimulating the tumor, αv and α2 integrin subunits, in the presence of CTX, resulted in inhibition of endothelial cell projection, presenting a round shape and membrane ruffles with actin marked these immature adhesions. The α2 subunit distribution in collagen and laminin coating exhibited lower intensity accompanied by more significant retraction of the cell body, without the regular formation of protrusions; this characteristic corroborated the data obtained in the wound healing assay with tumor stimulation, which inhibited migration by 81%.

Binding of integrins to their ligands in the extracellular matrix changes the endothelial cell cytoskeleton conformation, and consequently, cell migration. This is modulated by intracellular signaling pathways like focal adhesion kinase (FAK), leading to activation of Rho GTPases proteins and phosphorylation of myosin light chain, responsible for cell contractility (Bryan and D’Amore, 2007; Bloom and Zaman, 2014). Inhibition of Rac1 stimulates RhoA activity (implicated with SFs), thus inhibiting the formation of lamellipodia and filopodia (structures associated with the cytoskeleton projections on the front and rear parts of the cell, respectively) (Bloom and Zaman, 2014; Burridge and Guilluy, 2016). The development of lamellipodium protrusions is dependent on the rapid polymerization of actin in filaments. The 2/3rd protein complex related to actin (Arp2/3) is a crucial regulator of this process, which is responsible for the nucleation of new actin filaments by providing the necessary force for membrane protrusion to enlarge the existing filaments (Pollard and Borisy, 2003; Pollard 2007; Burridge and Guilluy, 2016). Also, marked inhibitory activity of CTX (50 nM) on Rac1 and FAK expression and the Arp2/3 complex in endothelial cells was observed, which explains the inhibitory action on the cytoskeleton morphology and dynamics evidenced in immunofluorescence, Western Blotting, and time-lapse assays performed in this study.

CTX inhibited VEGF secretion in t.End.1 cells, irrespective of whether t.End.1 cells were incubated in the presence of culture medium or stimulated with tumor conditioned media. Decreased CTX secretion of VEGF may be a consequence of FAK inhibition as VEGF, a signaling molecule, is involved in the production of this mediator (Wary et al., 2012). Just as decreased VEGF secretion contributes to the inhibition of α2 subunit expression, and αv in particular, besides the fact that this mediator activates these integrins during angiogenic and lymphangiogenic processes (Avraamides et al., 2008), it may, therefore, generate negative feedback. Thus, the inhibitory action of CTX on FAK expression is crucial for the diminution of endothelial cell events associated with angiogenesis. These results, along with those obtained in immunofluorescence analyses, such as morphology, cytoskeleton polymerization, and cell extension, are strongly correlated with FAK inhibition, protein kinase capable of stimulating actin polymerization, and filopodia formation involving regulation of the proteins from the Rho GTPases family (Schlie-Wolter et al., 2013; Hohmann and Dehghani 2019). This hypothesis is based on data from previous studies that demonstrated that CTX could inhibit the expression of Rho GTPases in both the macrophages, inhibiting the translocation of RhoA and Rac1, interfering with the cytoskeleton efficiency in capturing particles to be phagocytized (Sampaio et al., 2006b). In tumor cells of the WRC 256 strain, CTX inhibited RhoA and FAK kinase (Faiad et al., 2008), thereby compromising the actin filament polymerization of these cells involved in the proliferation and adhesion of the tumor cell. As future perspectives, *in vivo* studies using tumor models will be necessary to confirm the mechanisms involved in CTX-induced inhibitory effect on endothelial cells functions, specially on the emergence of new vessels.

## Concluding Remarks

**FIGURE 7:**
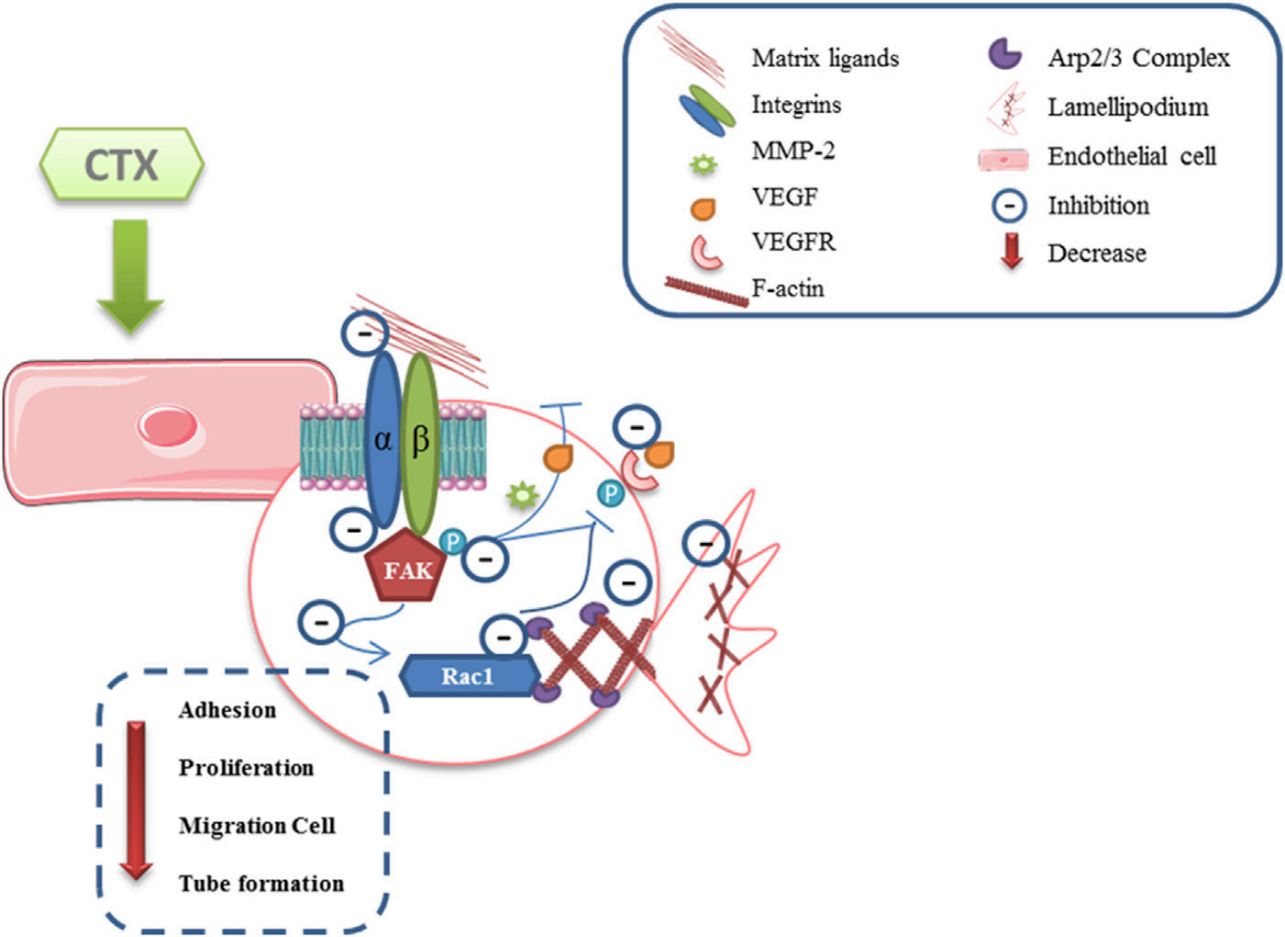
Scheme proposed for anti-angiogenic effect directly induced by CTX. CTX exerts its inhibitory effect by decreasing endothelial cell adhesion to their matrix ligands and, consequently, interferes with FAK kinase phosphorylation, which may lead to the inhibition of Rho GTPases Rac1, since these effector proteins activate the regulation of cell proliferation, migration, and invasion. Moreover, the inhibitory action on signaling molecules such as FAK may lead to a significant decrease in the secretion of critical mediators for the development of angiogenesis, MMP-2, while reducing speed and persistence of migration in 3D matrices, and of the VEGF, in turn decrease the stimulus on the same receivers. Furthermore, both of inhibited αv and α2 subunits of the integrins, and the decrease in VEGF binding to VEGFR lead to the inhibition of the Arp2/3 complex, a key regulator in actin polymerization and stress fiber formation (Scheme based in Lamalice et al., 2015). Supplementary Material should be uploaded separately on submission, if there are Supplementary Figures, please include the caption in the same file as the figure. Supplementary Material templates can be found in the Frontiers Word Templates file.

The present study showed that CTX inhibits cell adhesion on different extracellular matrix components (Figure 7). This inhibition was related to the reduction of αv and α2 integrin distribution, and cytoskeletal actin polymerization (F-actin), accompanied by inhibition of FAK, Rac1 (GTPase) signaling proteins. This was because these effector proteins activate the regulation of cell proliferation, migration, and invasion (Bryan and D’Amore, 2007; Hohmann and Dehghani, 2019). Furthermore, FAK inhibition induces a decrease in MMP-2 and VEGF secretion and MMP-2 and MMP-9 expression, while reducing the speed and persistence of migration in 3D matrices (Kim et al., 2008) and also inhibits the Arp 2/3 complex, an essential regulator for the polymerization of actin and the formation of lamellipodia (Lamalice et al., 2007). This study is the first to describe the direct inhibitory action of CTX on the critical events involved in angiogenesis and, therefore, contributes significantly to increasing knowledge on the mechanisms involved in the antitumor action of this toxin.

## Data Availability

The original contributions presented in the study are included in the article/Supplementary material, further inquiries can be directed to the corresponding author/s.
